# 2D Materials Boost Advanced Zn Anodes: Principles, Advances, and Challenges

**DOI:** 10.1007/s40820-023-01021-9

**Published:** 2023-02-08

**Authors:** Songhe Zheng, Wanyu Zhao, Jianping Chen, Xiaoli Zhao, Zhenghui Pan, Xiaowei Yang

**Affiliations:** 1 School of Materials Science and Engineering, Tongji University, Shanghai, 201804 People’s Republic of China; 2 School of Chemistry and Chemical Engineering, Shanghai Jiao Tong University, Shanghai, 200240 People’s Republic of China

**Keywords:** Zinc-ion battery, Large-scale energy storage application, Zn anode, Lifespan, 2D materials

## Abstract

Aqueous zinc-ion battery (ZIB) featuring with high safety, low cost, environmentally friendly, and high energy density is one of the most promising systems for large-scale energy storage application. Despite extensive research progress made in developing high-performance cathodes, the Zn anode issues, such as Zn dendrites, corrosion, and hydrogen evolution, have been observed to shorten ZIB’s lifespan seriously, thus restricting their practical application. Engineering advanced Zn anodes based on two-dimensional (2D) materials are widely investigated to address these issues. With atomic thickness, 2D materials possess ultrahigh specific surface area, much exposed active sites, superior mechanical strength and flexibility, and unique electrical properties, which confirm to be a promising alternative anode material for ZIBs. This review aims to boost rational design strategies of 2D materials for practical application of ZIB by combining the fundamental principle and research progress. Firstly, the fundamental principles of 2D materials against the drawbacks of Zn anode are introduced. Then, the designed strategies of several typical 2D materials for stable Zn anodes are comprehensively summarized. Finally, perspectives on the future development of advanced Zn anodes by taking advantage of these unique properties of 2D materials are proposed.

## Highlights

- The mechanisms of two-dimensional (2D) materials protecting Zn anodes were summarized.
- The recent progress of 2D materials boosting advanced Zn anodes was exhaustively categorized and reviewed.
- The prospects in the commercial application of aqueous zinc ion batteries were discussed.

## Graphical abstract

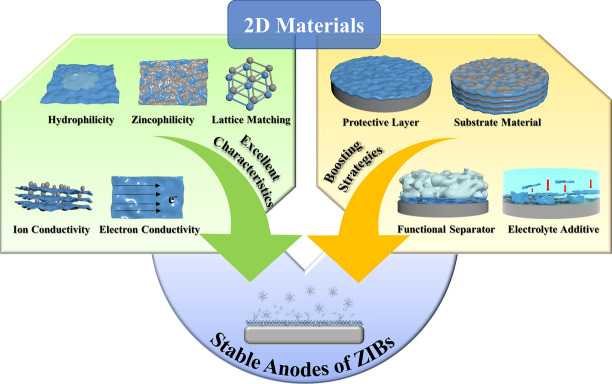

## Introduction

With the increasingly growing energy demands and the depletion of traditional fossil fuels as well as the corresponding environment pollution [1–4], the development of clean and renewable energy resources (such as solar, wind, and tidal) has significantly stimulated the enthusiastic of researchers [5, 6]. Therefore, advanced energy storage systems (ESS) are urgently necessary as the medium to address the issues related safe and continuous energy harvesting and storage [6–13]. Among them, lithium-ion batteries (LIBs) have dominated the applications of secondary batteries and achieved great progress in the last 30 years [14, 15]. However, the increasing concerns on the current design of LIBs, such as limited lithium reserve, high cost, unsafe nature, and environmental issue [16], have prompted the search for alternative ESS. Although cost issue can be alleviated by developing sodium-ion batteries (SIBs) or potassium-ion batteries (PIBs) based on cheaper sodium or potassium metal anode, the safety and environmental concerns are difficult to be further addressed [17, 18]. To this end, aqueous batteries using high safety and eco-friendliness aqueous electrolytes, especially for aqueous zinc-ion batteries (ZIBs), can well meet these requirements due to the abundant reserve, low cost, high theoretical capacity (820 mAh g^−1^), and low redox potential (− 0.76 V vs. standard hydrogen electrode) of Zn metal anode [16, 19–29].

Recently, great efforts have been devoted to exploring high-performance cathodes of ZIBs, such as manganese oxides [30–34]-, vanadium oxides [35–38]-, and Prussian blue analogues (PBA) [39–44]-based cathodes. By taking advantage of various structural modifications and a series of mechanism studies, ground-breaking advances on the above-mentioned cathode materials have been achieved. Nevertheless, the capacitance, cycle life, and safety of ZIBs are significantly degraded by the Zn anode issues, including Zn dendrites and corrosion, thus restricting their further application [45]. Moreover, the intrinsically thermodynamic-driven reaction between water and Zn metal might bring about a hydrogen evolution reaction (HER) [46–49], which will compete with the stripping/plating of Zn, leading to low coulombic efficiencies and produce gas to cause electrolyte leaking [50–53]. Therefore, the development of efficient protective strategies or the design of suitable materials for advanced Zn anodes of ZIBs is necessary.

Since the discovery of graphene a few decades ago, a growing number of two-dimensional (2D) materials have gained enormous attention in energy storage [54–58] due to their unique chemical, physical, and mechanical properties. Indeed, 2D materials, as a class of ultrathin layer-structured nanomaterials, possessing ultrahigh specific surface area, much exposed active sites, superior mechanical strength and flexibility, and unique electrical properties [59–63] have been widely demonstrated to realize advanced Zn anodes for ZIB. However, a comprehensive and detailed review of the design principle, research advance and future challenge of 2D materials toward stable Zn anode for high-performance ZIBs has not been published so far.

**Fig. 1:**
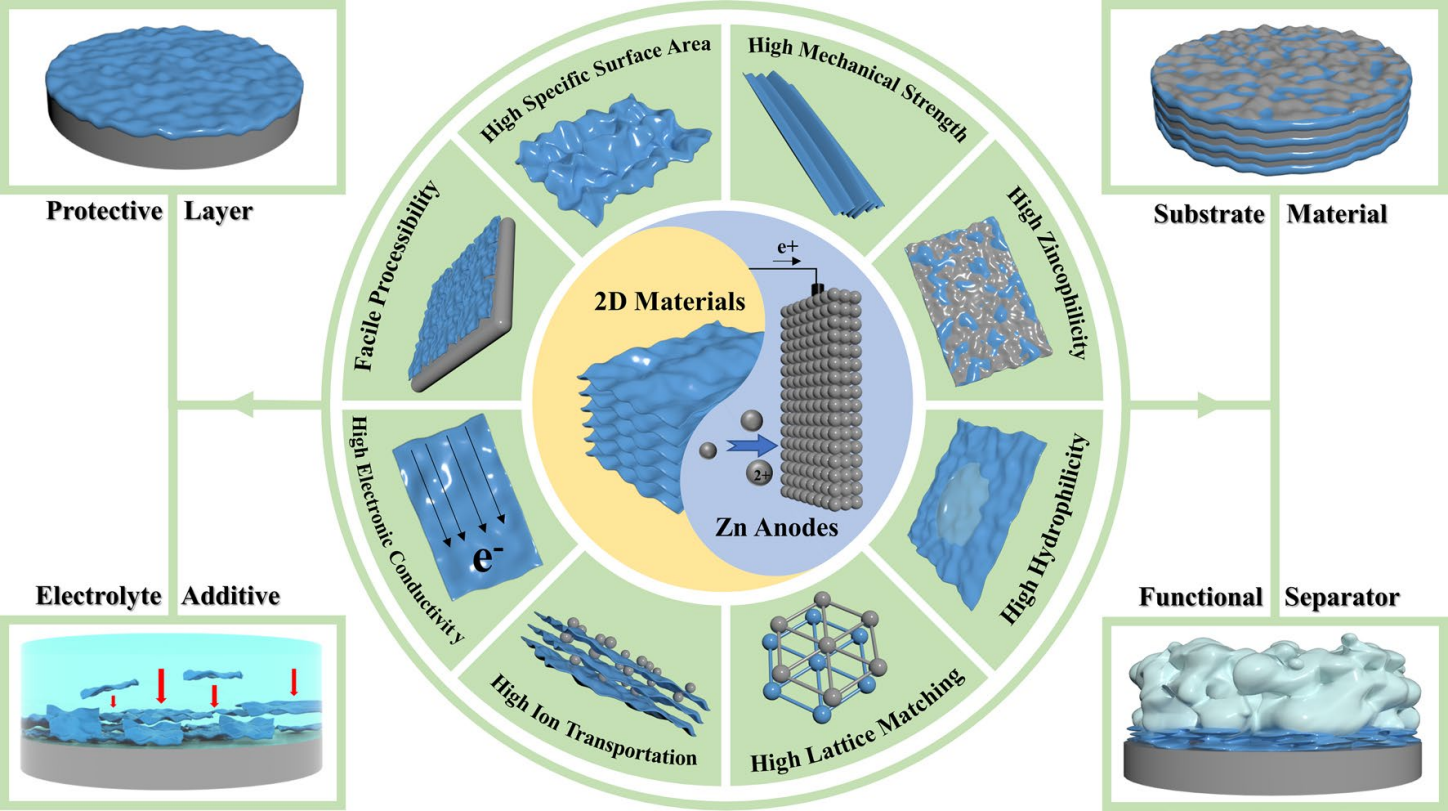
Overall schematic diagram of harnessing the unique properties of 2D material toward advanced Zn anodes for high-performance ZIBs

In this review, we will go over the latest advances for the application of several typical 2D materials on advanced Zn anodes from the perspective of serving as the protective layer, host material, electrolyte additive, and functional separator (Fig. 1). Initially, the fundamental principles of 2D materials against the drawbacks of Zn anodes are introduced. Subsequently, the designed strategies of 2D materials in improving the electrochemical performance of Zn anodes are summarized and discussed comprehensively. In the end, perspectives on the future development of high-performance Zn anodes by taking advantage of these unique properties of 2D materials are proposed.

## Fundamental Principles of 2D Materials Against the Issues of Zn Anodes

Zn metal is an ideal anode for aqueous ion batteries because of its high overpotential for hydrogen evolution. However, the Zn anode has inherent defects, which lead to severer issues such as Zn dendrites, corrosion, and passivation. Attributed to their excellent characteristics (Fig. 1), 2D materials have been demonstrated to improve the overall performance of ZIBs by handling these Zn anode problems. In this section, insights into the origins of key issues pertaining to Zn anode and mechanism understanding on how 2D materials reinforce the cycle stability and the safety performance of Zn anode will be discussed.

### Issues Facing Zn Metal Anodes

Generally, Zn anodes of ZIBs with aqueous electrolytes are expected to undergo a highly reversible Zn^2+^ plating/stripping process. However, electrical fields on the surface of the bumpy commercial Zn foil are usually uneven due to the “lighting rod effect” verified by Gauss’ law, which will lead to a localized deposition of Zn^2+^, facilitating the formation of vertically erected Zn dendrites [45, 47, 50–53]. The Zn dendrites can not only degrade battery performance but may pierce through the separator and make contact with cathodes, resulting in a short circuit of the battery. Apart from the Zn dendrite issue, HER mainly caused by water in the electrolyte [48] leads to low coulombic efficiency (CE) and produces gas to cause electrolyte leaking [52]; meanwhile, the generated OH^–^ may combine with SO_4_^2−^ and Zn^2+^ in the electrode/electrolyte interface and form Zn_4_SO_4_(OH)_6_·*n*H_2_O byproduct [46, 47]. Note that the dendrite growth and side reactions (HER and corrosion) have a clear inner relationship and will promote each other to aggravate the ZIBs’ performance. Therefore, it is necessary to consider their internal relations for developing efficient protective strategies or designing suitable materials for advanced Zn anodes of ZIBs.

### Principles of 2D Materials Reinforcing Zn Anodes

**Fig. 2:**
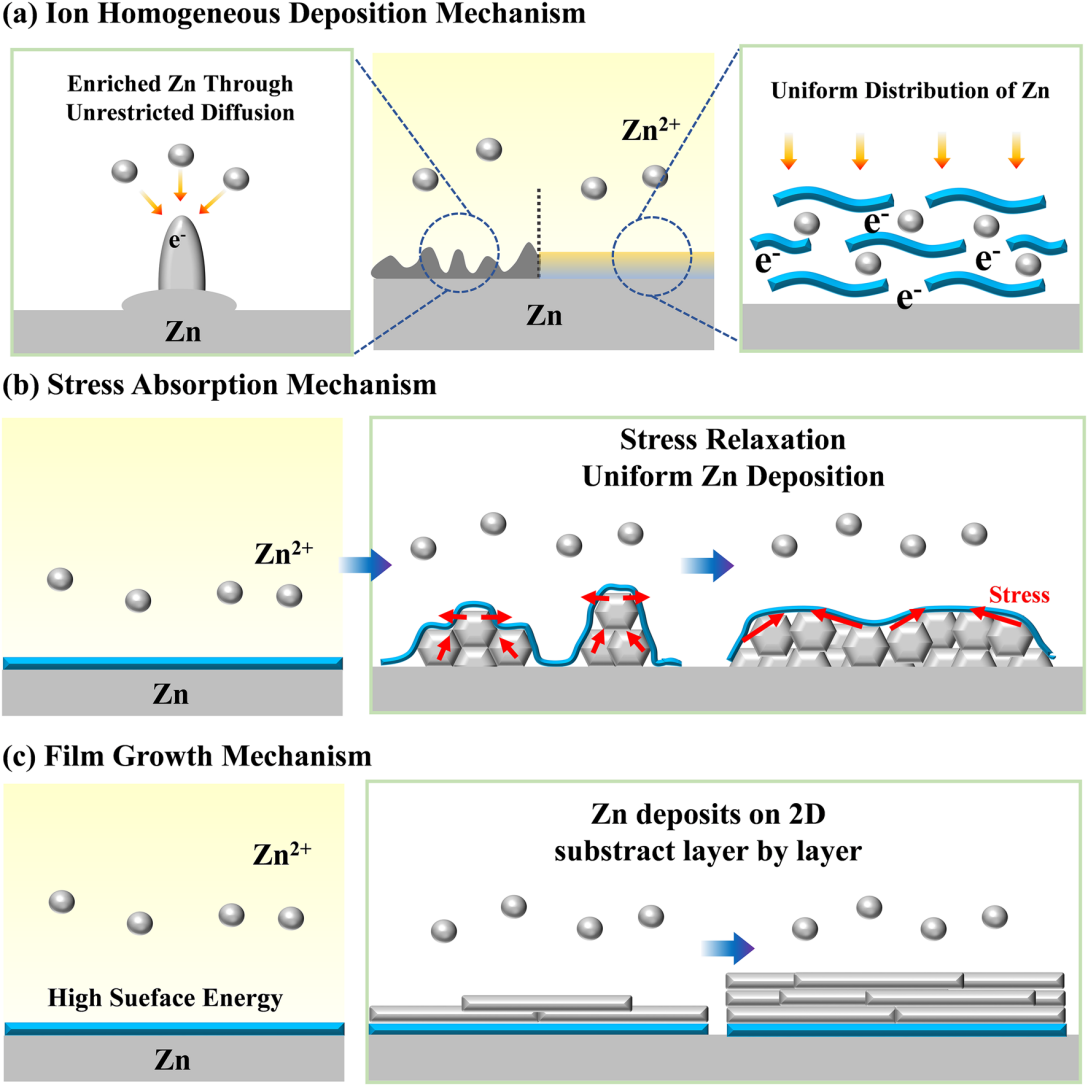
Protective mechanisms of Zn anode based on 2D materials. **a** The interphase formed by 2D materials has high conductivities of electron and ion, which successfully disperse particles to reduce the concentration polarization and postpone the dendrite occurrence. **b** Protective layer with high mechanical strength can efficiently release the stress generated during Zn deposition process. **c** 2D-nanosheets-formed interphase with higher surface energy makes Zn inclined to deposit on substrate layer by layer

Through the analysis of the issues of Zn anodes, it can be deduced that the dendrite growth is the most outstanding challenge urged to be settled. Generally, 2D materials protect anodes can be explained by three mechanisms (Fig. 2, Table 1) [64–66], including ion homogeneous deposition mechanism, stress release mechanism, and film growth mechanism. Moreover, the established theoretical models of dendrite can help us understand the mechanisms of how 2D materials improve the overall performance of ZIBs, which will be introduced as follows.

**Table 1:** Theoretical models of dendrite growth and corresponding strategies using 2D materials

Model	Mechanism	Strategy with 2D materials	Refs
Diffusion model	Space charge formation	Dissipate the current density/accelerate the ions mobility	[64]
Plating model	Surface tension release	Physically blocking	[67]
Film growth model	Interfacial energy	Fabricate high surface energy substrate	[65]

Chazalviel’s diffusion model [64] is a classic kinetics model, which points out the dendrite growth occurred around “Sand’s time” (*τ*_*s*_) (shown as Eq. 1), corresponding to the ion depletion in the vicinity of the negative electrode, especially at high current density. During the charging process, the cation is rapidly consumed, after which its concentration near the electrode surface is expected to drop to zero at the moment in time. Afterwards, a strong electronegative electric field absorbs and deposits a large number of cations in a short period of time, causing the growth of dendrites. This behavior is known as "Sand behavior," and this time is called Sand’s time:

1 $$ \tau_{s} = \pi D\frac{{\left( {eC_{0} } \right)^{2} \left( {\mu_{a} + \mu_{{Zn^{2 + } }} } \right)^{2} }}{{\left( {J\mu_{a} } \right)^{2} }} $$

where *D* represents the ambipolar diffusion constant, *e* is the electronic charge, *C*_*0*_ is the initial concentration of the electrolyte, ${\mu }_{a}$ and ${\mu }_{{Zn}^{2+}}$ are the mobilities of anions and Zn ions, respectively, and *J* is the effective current density. For postponing dendrite occurrence (Fig. 2a), one viable plan is to homogenize the effective current density. By keeping the values of *τ*_*s*_ on the anode surface consistently, Zn ions can simultaneously deposit in different regions, keeping pace with each other and thus achieving uniform deposition [68]. Therefore, conductive 2D materials are suitable to confine Zn metal and disperse the effective current density (*J*) to a uniform value, along with the optimization of structural parameters, including morphology, size, thickness, and pore to facilitate longitudinal transport and limit lateral plane diffusion of Zn^2+^. Another strategy is to utilize the ion transport characteristic of 2D materials as electrolyte additives or to fabricate “soft” artificial protection on the anode surface. Edge/surface functionalization or crystal phase transition for 2D materials can accelerate the ion mobility near the anodes, thus reducing the concentration polarization and facilitating the uniform deposition of the plane [65].

Yamaki’s plating model [69] confirms that the formation of dendrites is a form of mechanical stress release [70–72]. The required pressure difference (*ΔP*) on the Zn surface is related to both the tension (*γ*) and the orthogonal curvature radius (*R*_*1*_*, R*_2_) of the surface, which is shown in Eq. 2:

2 $$ \Delta P = {\upgamma }\left( {\frac{1}{{R_{1} }} + \frac{1}{{R_{2} }}} \right) $$

To guarantee the process of dendrite growth, *ΔP* should exceed the creep strength of bulk Zn, forcing the newly grown Zn crystal nuclei and continually get bigger and bigger (Fig. 2b) [73]. By fabricating a “harder” barrier on the surface of Zn metal between anodes and electrolytes, like 2D nanosheets and their complex, dendrite puncture can be blocked obviously. Harder barriers absorb the stress released while the dendrites formation process, restricting *ΔP* at a level much lower than the creep strength of Zn, successfully limiting the continued growth of crystal nuclei [74, 75].

The film growth model [65] is to address the dendrite issue via the surface energy because this issue can be considered as a kind mode of film growth. Based on the capillarity of homogeneous nucleation, solid nucleate forms a prior unstable liquid by establishing a solid–liquid (s–l) interface. The film growth depends on both the surface energy difference between the substrate and the film and the lattice matching between the growth substrate and growing film. Since the matching degree mostly depends on the material itself and is hard to adjust, surface energy shall be the key factor, we can see from Eq. 3:

3 $$ \gamma_{{{\text{sub}}}} + Q_{e} = \gamma_{{{\text{Zn}}}} $$

where *Q*_*e*_ stems from the partial energy of the electric field. The smaller *γ*_sub_ becomes, the larger *Q*_*e*_ shall be needed, then leading to a more significant trend of dendrite growth. By transforming 2D nanosheets with large specific surface areas and abundant surface chemistry into an anode protective layer, it’s easy to get a high surface energy substrate to deal with the dendrite growth issue.

As mentioned above, the researches of 2D materials protecting Zn anodes mainly focus on four aspects, including protective layers, host materials, electrolyte additives, and functional separators. The ion homogeneous deposition mechanism takes effort in almost every strategy to uniform Zn^2+^ dispersion. In comparison, the stress absorption mechanism can help researchers understand why 2D-material-formed-interphase can limit dendrite-continued growth. When 2D materials work as host materials, especially substrates, Zn deposits along the plain, which can be explained by the film growth mechanism.

## 2D Materials for Advanced Zn Metal Anodes

**Fig. 3:**
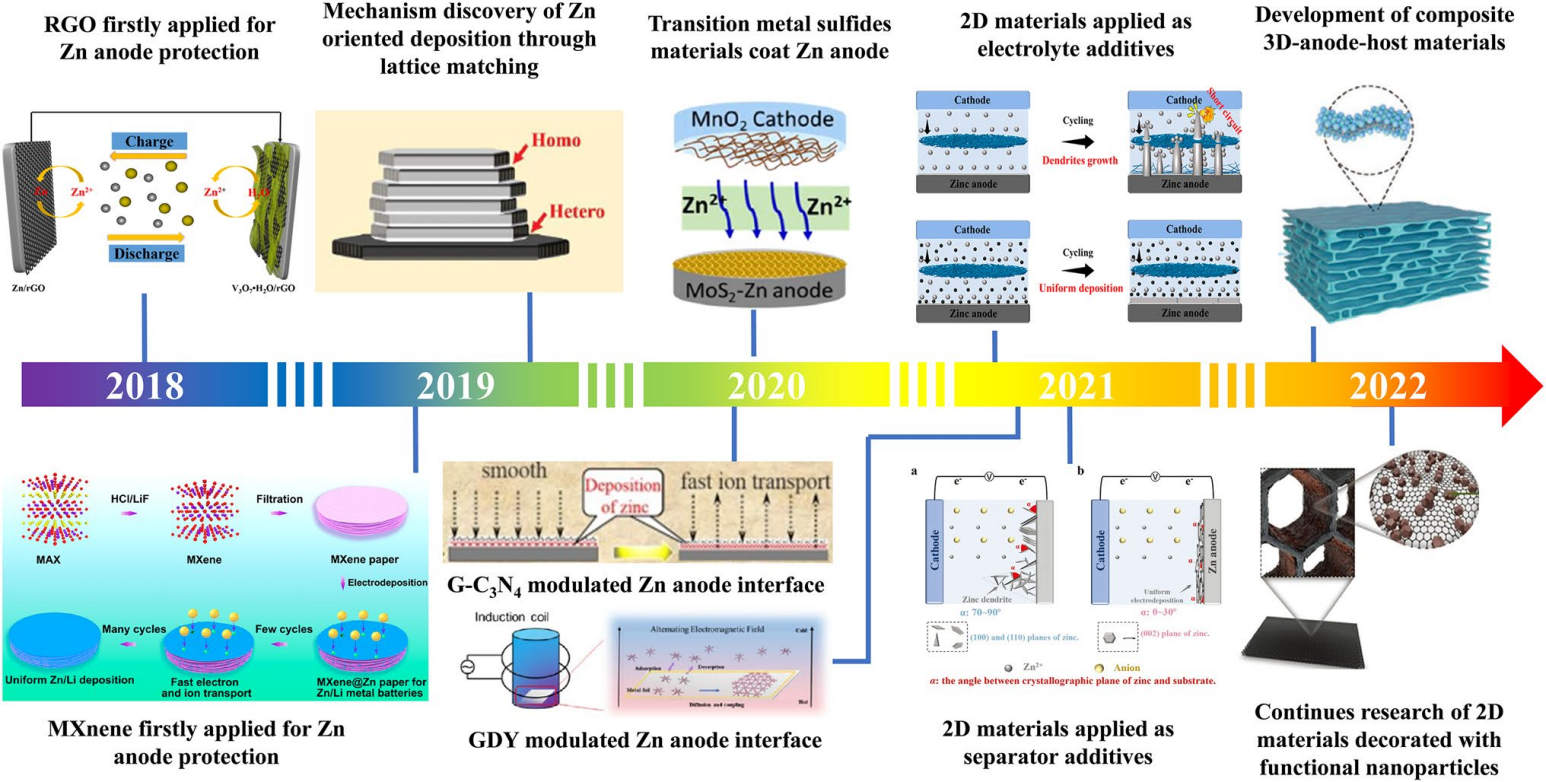
A brief timeline summarizing the development of 2D materials in Zn anodes of ZIBs. In 2018, Zn firstly coated with 2D material as the anode of ZIBs. Copyright 2018, American Chemical Society. In 2019, mechanisms for 2D materials reinforcing Zn anodes have been deeply researched. Copyright 2019, American Chemical Society; Copyright 2019, American Association for the Advancement of Science. In 2020, other 2D materials starting to be applied in Zn anode protection. Copyright 2020, American Chemical Society; Copyright 2020, Elsevier; Copyright 2021, IOP. In 2021, 2D materials have been applied as electrolyte additives and separator additives. Copyright 2021, American Chemical Society; copyright 2021, Wiley. In 2022, multi-functional modified 2D materials have been applied as anode-hosts. Copyright 2022, Wiley

As shown in Fig. 3, the primary attempt at 2D materials applying in ZIBs can date back to 2018, Zn coated with reduced graphene oxide (RGO) as the composite anode of ZIBs [76]. Subsequently, deeper research about mechanisms for graphene reinforcing Zn anode stability was explored in 2019 [77]. Since then, different types of 2D materials and different strategies in Zn anode protection have been developed. Aside from working as an anode protective layer or deposition framework, researchers have also explored the possibility of 2D materials for electrolyte additives or separator modifiers [74, 75, 78–80]. On the other hand, in order to further enhance the practicality of 2D materials and expand their applicability, various kinds of molecules, zero-dimensional (0D) particles, or other nanomaterials and polymer materials have been recently tried to form composite with 2D nanosheets, achieving an improved ZIB performance [80–97].

### Graphene for Advanced Zn Anodes

Carbon has a large number of allotropes with different properties, which has attracted extensive attention in the research community. Graphene was discovered at the beginning of this century. It has a unique 2D planar structure, and its covalent *π*-conjugated honeycomb plane consists of pure *sp*^2^ hybridized carbon, which enables many excellent properties, including acceptable structural stability and ultrafast electron transfer [98]. At the same time, graphene can also be regarded as the parent of all other carbon allotropes, except the diamond with a three-dimensional regular tetrahedron structure formed by *sp*^3^ carbon. These carbon allotropes are produced by graphene through different folding and stacking methods. In addition, graphene has also promoted the discovery of many other new 2D materials, such as 2D metal carbides and nitrides (MXenes), transition metal disulfides (TMD), and 2D perovskite materials.

Graphene has an excellent performance in a number of characteristics, such as large specific surface area (2600 m^2^ g^−1^), admirable electronic conductivity (10^7^ S m^−1^), good light transmittance (97.7%), high mechanical strength (with Young's modulus of ≈ 1.0 TPa), and high thermal conductivity (5300 W m^−1^ K^−1^) [98]. Because of these unique properties, graphene has great application potential in many fields, especially for energy storage like ZIBs. Strong mechanical properties efficiently release the crystalline stress in the plating Zn anode that inhibits the fast growth of substantial Zn dendrites, while high electronic conductivity effectively enables uniform charge distribution, leading to a smooth Zn^2+^ deposition. These properties render graphene an ideal material applied for the protection of the Zn metal anode by inhibiting the formation and growth of dendrites.

**Fig. 4:**
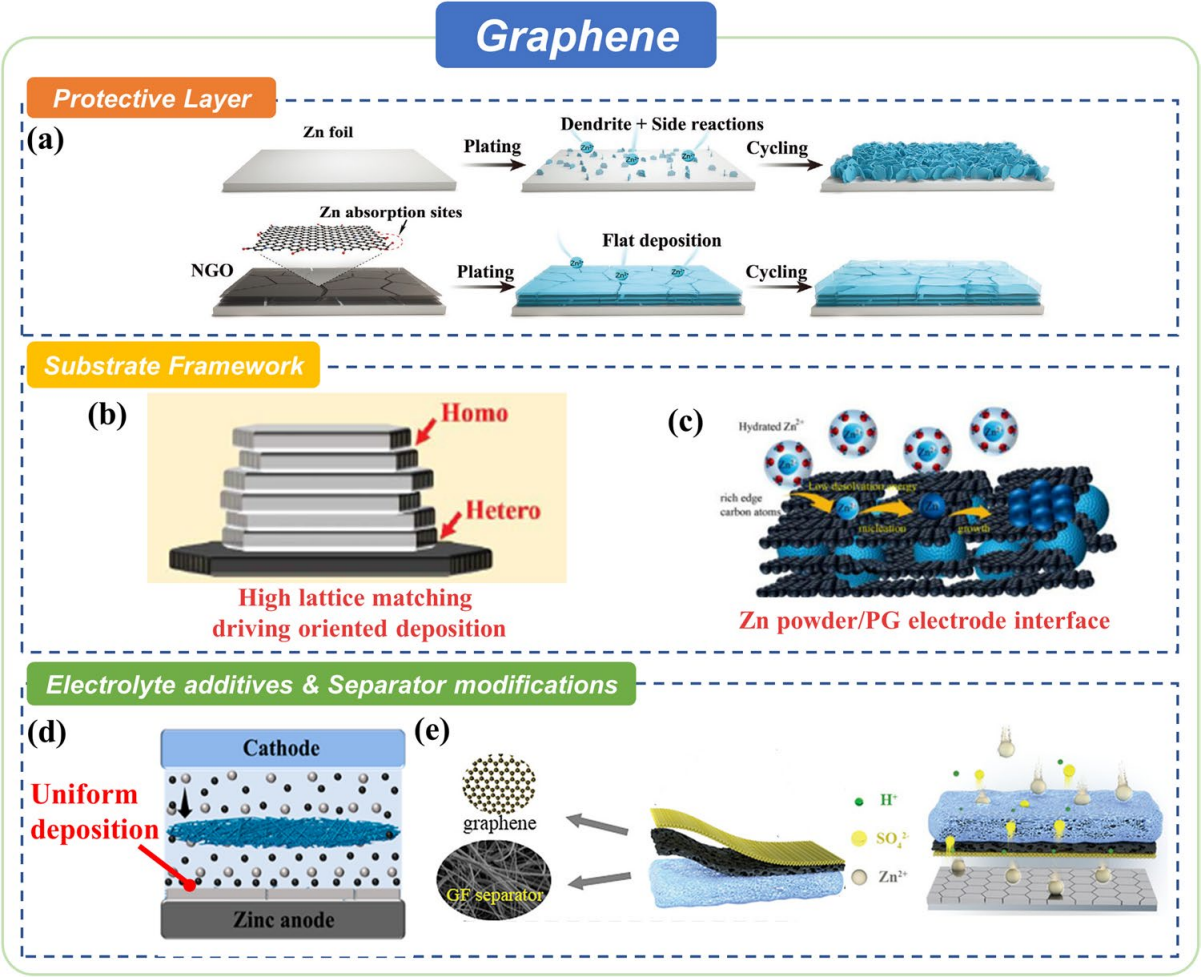
Summary of graphene strategies for advanced Zn anodes. **a** Ultrathin surface coating of NDG. Copyright 2021, Wiley. **b** Confirmation of the relationship between Zn deposition and crystallographic orientation through graphene materials. Copyright 2019, American Association for the Advancement of Science. **c** 3D pristine graphene network combined with Zn powder. Copyright 2022, Elsevier. **d** GO electrolyte additive. Copyright 2021, American Chemical Society. **e** A functional separator constructed via parallelly grown graphene sheets. Copyright 2022, Wiley

Aside from pure graphene, researchers also developed modified graphene for Zn anode protection, such as graphene oxide (GO) containing oxygen functional groups and nitrogen-doped graphene oxide (NGO) embedded by N elements. These derivates utilize their hydrophilicity, zincophilicity or size effect to facilitate rapid and smooth deposition of Zn^2+^ [76, 86]. As for HER and other side reactions, these materials not only restrict them by limiting the continuous growth of the contact area between Zn and H_2_O during charge–discharge processes but also by destroying the hydration shell of Zn^2+^ with zincophilic functional group to reduce the activity of water molecules. This section will summarize the Zn anode protective methods, including surface engineering, host architecture, separator modification, and electrolyte additives, through the application of graphene and their derivates (Fig. 4).

#### As Protective Layers

Solid electrolyte interphase (SEI) is formed between the anode and electrolyte during the initial charge–discharge process. It has the disadvantage of inhomogeneous distribution and brittle mechanical strength, which might deteriorate the dendrite growth and side reactions in ZIBs. Building artificial SEI as Zn anodes protective layers is a practical method to get away from those undesirable phenomena. A rational design for this kind of surface engineering requires those characteristics, including proper thickness, high stability in battery systems during cycling, well interfacial contact, along with improvements in ion transportation and physical strength.

Zn anodes protective layers fabricated by graphene and its derivates can be considered a kind of SEI to some extent, dealing with their 2D feature (easy forming protective layers with proper thickness), high stability, hydrophilicity, zincophilicity, and other characteristics. Their function of inhibiting dendrite growth and side reactions has already been employed in research for years. Wei and co-workers first utilized rGO in both ZIB anodes and cathodes due to its high electronic conductivity and excellent mechanical properties [76]. The rGO protective layer can effectively inhibit the Zn dendrite formation by enabling a uniform charge distribution and providing fast and effective electron transfer channels. Besides, the rGO layer restrains the dendrite formation through its mechanical properties while strongly attached to the Zn metal surface.

Zincophilicity is another proper characteristic aside from electronic conductivity that can increase the application potential of graphene derivatives. Chen et al. (Fig. 4a) used NGO to fabricate a parallel and ultrathin interface modification layer on Zn foil [86]. The NGO is one step synthesized by a Langmuir–Blodgett method. The parallel graphene layer and zincophilic N/O elements achieved the directional deposition of Zn crystal in the (002) planes. This kind of deposition formation successfully avoids the formation of dendrites on the surface of Zn metal. Meanwhile, the ultrathin NGO layer at the interface improves the diffusion kinetics of Zn^2+^ and suppresses the side reactions of HER and byproduct generation in mildly acidic electrolytes through insulating metal and electrolytes.

Aside from tuning the composition of graphene, another feasible method is to introduce other useful components to the compound to improve the protective properties of the graphene-containing layer. Qian et al. constructed a bifunctional cellulose nanowhisker-graphene (CNG) membrane to mitigate the uncontrollable dendrite growth and side reaction problems [99]. Simple mixing nanowhisker and graphene nanosheets can combine functions derived from these two materials. This research reveals that the CNG membrane functions as a desolvation layer to preclude H_2_O molecules from encountering the Zn anode, therefore retarding the water-induced side reactions like HER. This protective layer with negative surface charges can uniformly spread Zn^2+^ to obtain smooth Zn deposition. Furthermore, the flexible and toughened CNG membrane could withstand a strong tensile force and a great puncture force to favorably accommodate the Zn anode surface fluctuation during plating/stripping.

Till now, the composite protective layer method has been developed in the direction of integration. Recently, Liu et al. constructed a multi-functional protective layer (ZGL) which consists of zeolitic imidazolate framework (ZIF-8) decorated graphene oxide (GO) and PVDF to regulate the deposition behavior of Zn^2+^ on ultrathin Zn anode [100]. The ZIF-8 grown on GO nanosheets enhanced Zn^2+^ deposition property and homogenized ion flux to implement smooth deposition. At the same time, PVDF worked as a separator, allowing the protective layer to completely separate anodes and electrolytes and suppressing side reactions through separate anodes and electrolytes.

#### As Host Materials

Commonly, the excessively used metallic Zn anodes comprise a dominant share of the device weight. Thus, lighter anodes with proper capacity-matching cathodes are essential to achieve high energy density at the device level. An ideal solution is electrochemically depositing Zn with proper capacity on the substrates containing properties like lightweight and high electronic conductivity. Therefore, graphene can be a feasible material since its characteristics almost completely meet the demand for substrates.

In addition to the well-known features, graphene can deposit Zn metal in specific crystal planes, which was first discovered by Zheng and co-workers (Fig. 4b) [77]. They reported an epitaxial mechanism to regulate the nucleation, growth, and reversibility of metal anodes. Graphene was proved to be effective in driving the deposition of Zn with a locked crystallographic orientation relation due to its low lattice mismatch for Zn. In another work, Li et al. (Fig. 4c) developed a simple and effective route to process zinc powder into usable and high-performance zinc anode by applying multifunctional pristine graphene (PG) network [101]. The electrodes can be formed because of the macroscopic assembly property of PG sheets. Simultaneously, PG can enhance charge transfer and then facilitate the electrodeposition kinetics of zinc, and the unique hexagonal lattice structure can promote horizontally aligned zinc deposits.

Modifications for graphene containing functional groups or functional elements can be useful for stable and uniform Zn deposition. For example, Fu et al. effectively extended the cycle life of ZIBs by using a nanofluidic channel (NC) layer as a substrate, which was made up of poly(3,4-ethylenedioxythiophene)-poly(styrenesulfonate) (PEDOT:PSS) decorated graphene sheets (GSs) produced by electrochemical exfoliation [84]. The NC layer not only effectively modulated the Zn ion distribution density and inhibited dendrite growth but also restrained HER and other side reactions due to its surface functional groups applied by the polymer additives. Except for modifications containing functional groups or functional elements, nano-metal particles can be another kind of feasible composite material facilitating the rapid and uniform deposition of Zn.

The architecture of substrate materials contains not only the panel structure but also the 3D host structure, where Zn^2+^ ions have extensive contact areas and larger deposition space. Besides, high electronic conductive graphene with 3D porous structures makes electron and ions distribution more uniform. Yin et al. investigated hexagonal WO_3_/three-dimensional porous graphene (*h*-WO_3_/3DG) as intercalation anode materials for ZIBs [88]. The hexagonal pore canals of h-WO_3_, high specific surface area, and electrical conductivity of 3DG endow the *h*-WO_3_/3DG anode with fast reaction kinetics and structural durability. In addition, Chen et al. fabricated a 3D porous graphene-carbon nanotube scaffold decorated with metal–organic framework-derived ZnO/C nanoparticles (3D-ZGC) as the host for dendrite-free composite Zn anodes [92]. The zincophilic ZnO/C nanoparticles acted as preferred deposition sites with low nucleation barriers to induce homogeneous Zn deposition. Furthermore, the mechanically robust 3D scaffold with high conductivity not only suppressed the formation of dendritic Zn by reducing the local current density and homogenizing Zn^2+^ flux but also inhibited volume changes during the long-term plating/stripping process.

#### As Electrolyte Additives and Separator Modifications

Modified graphene, especially GO and rGO, can also be applied as additives for electrolytes. Taking advantage of the high dispersibility of graphene in the liquid phase due to electrostatic repulsion, as well as high affinity and fast transfer to Zn^2+^, to achieve uniform dispersion, fast transport, and smooth deposition of ions would be an effective strategy. Spontaneously reducing and assembling graphene on Zn foil through the redox reaction of oxide-containing groups and Zn facilitates the formation of a protective interface between anodes and electrolytes. Besides, the strong mechanical properties and high electronic conductivity can inhibit the continuous growth of Zn dendrites to avoid short circuits while cycling. For example, Qin et al. (Fig. 4d) synthesized a hybrid electrolyte with GO as an additive to promote the uniform distribution of the electric field and reduce the nucleation overpotential of Zn^2+^, displaying a smooth Zn electrodeposition layer and reaction kinetics [79].

As we all know, the separator plays a critical role in ZIBs, which facilitates uniform ion transport while blocking electron transfer and dendrite growth to avoid short circuits. A qualified separator might possess the following features: adequate porosity, good mechanical strength, favorable electrolyte wettability, and high ionic conductivity. Fortunately, modified graphene possesses almost all these features. Zhou et al. (Fig. 4e) developed a functional separator constructed via parallelly grown graphene sheets modified with sulfonic cellulose on the one side of the commercial glass fiber separator through the spin coating technique [102]. This separator can consistently regulate Zn growth toward a locked crystallographic orientation of Zn (002) texture to intercept dendrites. Furthermore, the separator can spontaneously repel SO_4_^2−^ and anchor H^+^ while allowing effective transport of Zn^2+^ to alleviate side reactions.

### Other Carbon-based 2D Materials for Advanced Zn Anodes

Apart from the most famous 2D carbon material graphene, other carbon-based 2D materials, including graphdiyne (GDY), graphitic carbon nitride and expanded graphite, have also been reported to eliminate the undesirable dendrite formation and side reactions through several strategies.

GDY is a 2D carbon allotrope other than graphene, in which benzene rings are conjugated by 1,3-diyne bonds to form a 2D planar network structure. GDY has abundant carbon chemical bonds, a large conjugated system, wide interplanar spacing, excellent chemical stability, and semiconductor performance. Its strong bond energy endows GDY with excellent flexibility and mechanical properties [103]. Moreover, the Young's modulus value of GDY can reach 470 GPa, which is very close to graphene and carbon nanotubes, indicating that GDY is an ideal self-supporting material capable of flexible layers.

Compared with graphene, GDY has a large number of pore structures with a diameter of 5.4 Å, which are uniformly distributed throughout the molecular structure, facilitating the diffusion and transfer of small ions and molecules. As the thickness of GDY decreases, its electronic conductivity and ion transportation increase gradually. Furthermore, the wide interlayer spacing of GDY is advantageous to ion transportation along the carbon plane of GDY [104]. All these properties guarantee the application advantages in the Zn anode protection field.

Expanded graphite (EG) is a kind of loose and porous wormlike material obtained from natural graphite scales through intercalation, washing, drying, and high-temperature expansion. In addition to the excellent properties of natural graphite, such as high electronic conductivity, EG also has a hollow structure, well absorption for electrolytes, good ion transportation, and other characteristics that natural graphite does not have [87]. These features hinder EG from becoming an easier fabricating anode material than a graphene membrane through stripping and stacking.

**Fig. 5:**
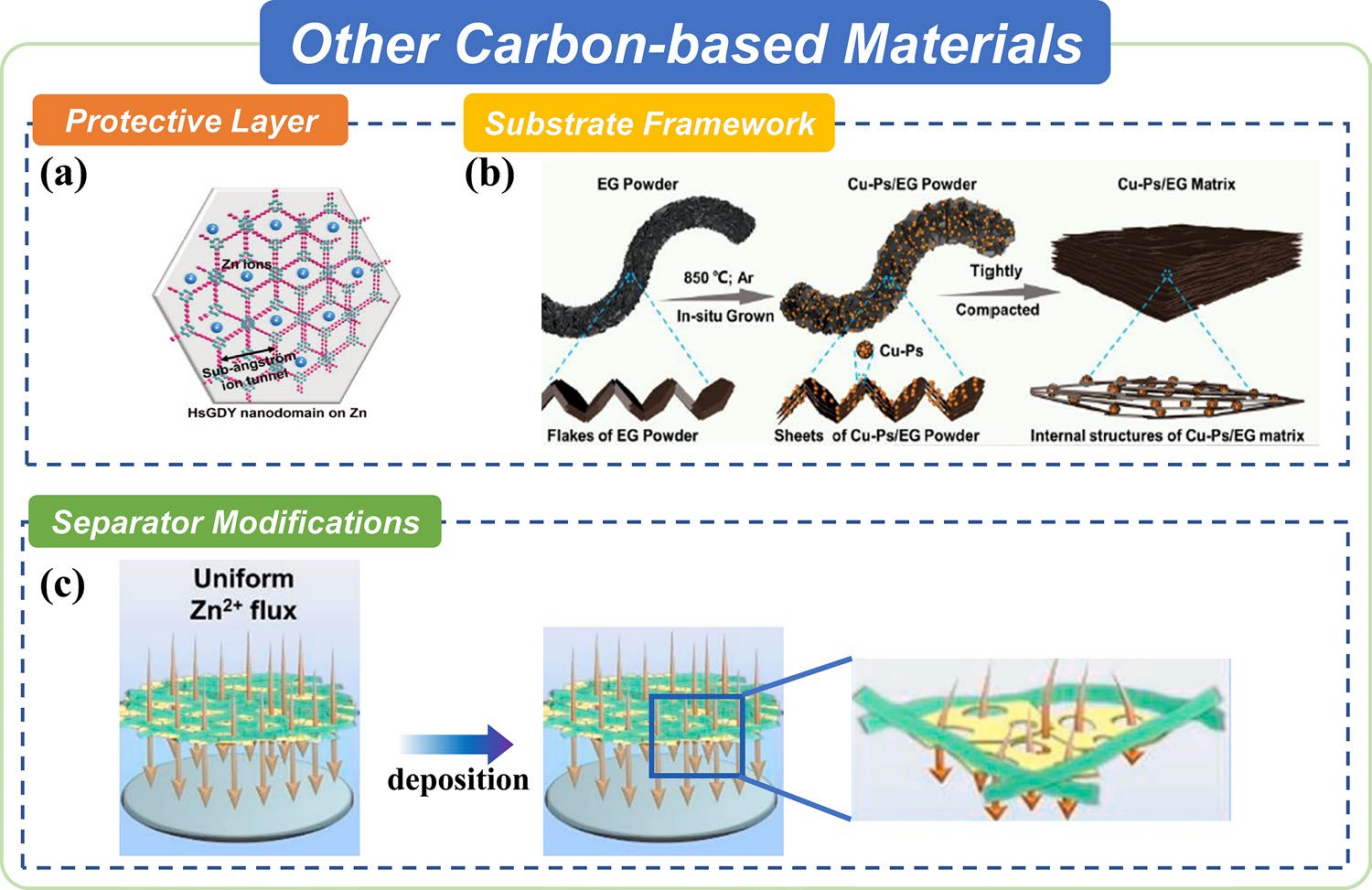
Summary of other carbon-based 2D materials for advanced Zn anode. **a** Hydrogen-substituted graphdiyne ion tunnels directed concentration redistribution. Copyright 2020, Wiley. **b** In-situ grown Cu particles on expanded graphite enabled dendrite-free and flexible Zn metal anode. Copyright 2022, Elsevier. **c** 2D porous g-C_3_N_4_ nanosheets manipulated Zn smooth deposition. Copyright 2021, Elsevier

Another feasible carbon compound for Zn anodes is graphitic carbon nitride (g-C_3_N_4_). The g-C_3_N_4_ is a planar 2D sheet structure similar to graphene, which has two basic units, respectively, using the triazine ring (C_3_N_3_) and 3-s-triazine ring (C_6_N_7_) to extend infinitely to form a network structure, and the 2D nanosheets are combined by van der Waals force [105–108]. The rich N element successfully improves the zincophilicity of g-C_3_N_4_, which attracted attention to Zn anode protection. In previous research, these three materials have already been used as protective layers, host materials and separator additives (Fig. 5).

#### As Protective Layers

GDY, as a kind of attractive layered 2D carbon nanomaterial, has been demonstrated as an artificial interface layer for uniform Zn deposition. In one of the pioneer works, Zhi et al. (Fig. 5a) synthesized hydrogen-substituted GDY (HsGDY) decorated Zn anode through an alkynyl-site cross-coupling reaction [104]. The ordered occupation of hydrogen atoms on meta-positions of benzene rings brings large hexatomic rings into the HsGDY networks. This sub-angstrom level structure, together with the micro-gaps between adjacent HsGDY microdomains, constitutes the ion tunnels for Zn^2+^ transportation, realizing dendrite-free Zn anodes. Moreover, Zhang et al. proposed another approach for synthesizing a few-layered GDY on the arbitrary metal substrate using a radio frequency (RF) heating-directed temperature gradient at the solid/liquid interface [103] The RF irradiation only heats the metal substrate but not the solvent; thus, the cross-coupling reaction occurs on the metal substrate, and the monomers remain stable in the bulk solution. Therefore, they obtained GDY film with homogenous morphology and an average thickness of 1–2 nm on the surface of the metal substrate and successfully avoided the formation of Zn dendrites due to the high electrical conductivity and uniform pore size.

Another effective material is g-C_3_N_4_ due to its zincophilicity. Liu et al. constructed a zincophile interphase based on the 3D-printed g-C_3_N_4_ modulating interface to concurrently achieve homogeneous zinc nucleation and a dendrite-free growth, where 3D printing is an effective technology to fabricate the porous and self-limiting 3D structure [105]. The unique 3D structure design is an ideal modulating coating, thus improving the cycling stability of the Zn anode. Our previous work developed a multifunctional protective layer consisting of g-C_3_N_4_ and PVDF [108]. This strategy not only results in homogenous nucleation because of zincophilic g-C_3_N_4_ but also relieves the hydrogen evolution reaction and the formation of by-products by blocking the direct contact of the Zn anode with the electrolyte.

#### As Host Materials

EG has served as the host material to homogenize charge distribution due to its intrinsic porous structure and high conductivity. Hou et al. (Fig. 5b) reported a dendrite-free and flexible anode material that is based on EG decorated with in situ formed copper particles (Cu-Ps) [87]. In this material, the EG substrate induces epitaxial electrodeposition of Zn for suppressing dendrite growth, while the Cu-Ps act as zincophilic seeds that reduce Zn nucleation overpotential. Moreover, the Cu-Ps nail the graphite nanosheets tightly and enhance the mechanical strength and flexibility of the Cu-Ps/EG composite.

#### As Separator Modifications

Aside from substrate materials, g-C_3_N_4_ has also been used as separator modifications. For example, Xue et al. (Fig. 5c) coated g-C_3_N_4_ nanosheets onto a commercial cellulose fiber separator via a simple drop-casting route [107]. The porous 2D g-C_3_N_4_ nanosheets act as ion redistributors to induce homogenous Zn ion flux; thus, the g-C_3_N_4_ coated separator enables a dendrite-free Zn deposition and improves the reversibility of Zn metal anodes.

### MXene for Advanced Zn Anodes

MXene is a new family of 2D materials first synthesized in 2011. These kinds of transition metal carbides, nitrides, and carbonitrides have the general chemical formula M_*n*+1_X_*n*_T_*x*_ (M represents early transition metal; *X* represents carbon or/and nitrogen; T represents surface terminations; *n* = 1 ~ 4) and have already been synthesized in over 30 discrete stoichiometric forms, while hundreds of new forms of MXenes have been predicted to be thermodynamically stable [109]. MXene can be simply fabricated throw selectively etching MAX compounds, which have the general chemical formula M_*n*+1_AX_*n*_ (M represents transition metal; A represents main-group element; *X* represents carbon or nitrogen; *n* = 1 ~ 4). Moreover, MAX compounds have self-lubricating, high toughness, electronic conductivity and similar lattice structure to Zn metal due to the unique nano-layered crystal structure and hexagonal close-packed structure [110]. After moving the A atoms away from MAX compounds, the reactant shall be transformed into 2D nanosheets, which is MXene. It inherits some structural characteristics of MAX compounds, having a lattice structure in common with Zn metal, strong physical properties, and high electronic conductivity.

**Fig. 6:**
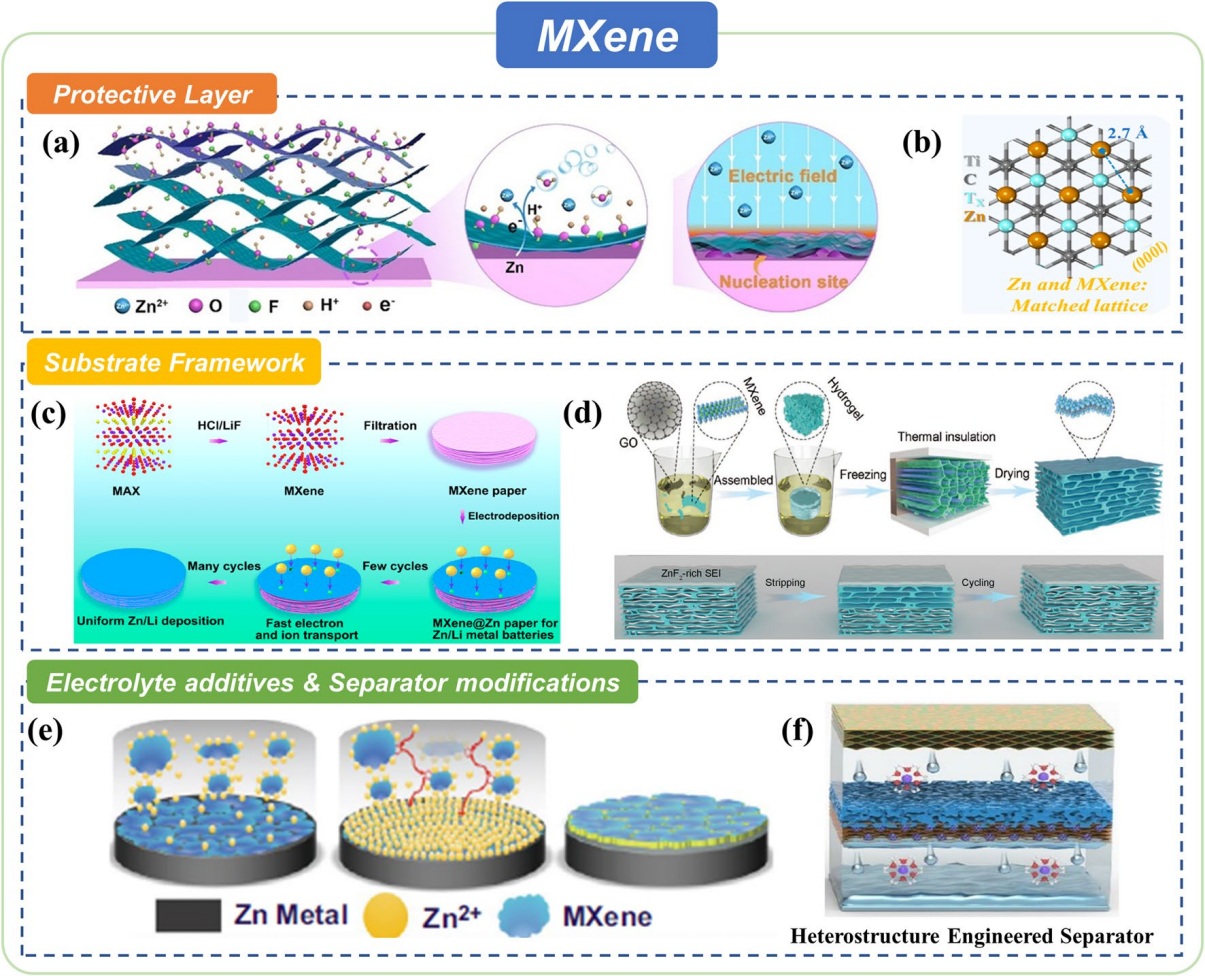
Summary of MXene strategies for advanced Zn anode. **a** Self-assembled MXene on Zn Anode. Copyright 2020, Wiley. **b** MXene with high lattice match to deposited Zn. Copyright 2021, American Chemical Society. **c** A freestanding MXene@Zn paper. Copyright 2019, American Chemical Society. **d** Hybrid MXene/praphene aerogel. Copyright 2021, Wiley. **e** Flexible MXene/nanocellulose hybrid film. Copyright 2022, American Chemical Society. **f** Zincophilic MXene/nanoporous oxide heterostructure engineered separator for ZIBs. Copyright 2022, American Chemical Society

Surface chemistry plays an important role in the physical and chemical properties of MXene, which is also an obvious difference compared with carbon-based 2D materials. The active transition metal atoms of MXene are exposed to the external environment, making the surface extremely easy to absorb functional groups, which is an obvious difference from graphene. These abundant and adjustable surface functional groups have an affinity with water and Zn^2+^. All these properties make MXene feasible in Zn anode protection. This kind of 2D material is anticipated to homogenize the local current on the surface between electrodes and electrolytes, boost the charge diffusivity, and promote uniform ion deposition [111]. In addition, it can also provide ample space for alleviating volume change and then alleviating the HER and other side reactions [112]. Compared with carbon-based materials ( such as GO and rGO), MXene shows high zincophilicity and hydrophilicity provided by the functional groups and high electronic conductivity, but its more complicated fabrication method limited the practical products as well. In this section, the progress in protecting Zn anodes employing the above-mentioned MXene properties will be summarized (Fig. 6).

#### As Protective Layers

MXene can be a kind of ideal material to fabricate the protective layer of the Zn anode due to its feasible thickness, strong intermolecular interaction with hydrophile, and zincophile characteristics. For example, Niu and co-workers developed a simple strategy to assemble the MXene layer on the surface of the Zn anode (Fig. 6a) [111]. Substantial oxygen-containing functional groups consist on the surface of MXene while the etching process. Therefore, the reduction potential of removing the oxygen-containing groups is higher than that of Zn/Zn^2+^ (− 0.76 V *vs.* SHE). Besides, functional groups make the surface of MXene negatively charged, which can be uniformly dispersed in the solution. The ultrathin and uniform protective layer can be fabricated according to these two characteristics through an in-situ spontaneously reducing/assembling strategy. The high electronic conductivity of MXene makes it feasible to modulate the local electrical field near the Zn surface. Meanwhile, surface functional groups with high zincophilicity decrease the nucleation energy barrier, resulting in uniform Zn deposition, enhancing cycle stability and cycle life. In another work, Zhi et al. reported a general and efficient molten salt etching method to adjust the species of halogen functional groups (Fig. 6b) [113]. Both DFT simulation and experimental characterizations identified that proper halogen functional groups improved lattice matching between MXene and Zn deposits, ensuring the formation of coherent heterogeneous interfacial regions at the early deposition stage, where Zn^2+^ preferred horizontal tiling to vertical stacking. Moreover, halogen functional groups played a guiding role in Zn^2+^ homogenous deposition.

Due to the strong intermolecular interaction between MXene films, the over-stacking of nanosheets may occur while constructing the protective layer. The introduction of spacers is a simple and easy solution. This method can also be used to introduce other functional materials into the protective layer to further improve battery performance. For example, Feng et al. introduced chitosan into MXene film by simply mixing these two dispersion liquids and coating them on the Zn foil [114]. Chitosan has hydroxyl and amine groups which can be served as coordination sites for transition metals. Meanwhile, electrostatic interaction can self-assemble this positively charged natural bio-polysaccharide on the surface of negatively charged MXene nanosheets. The protective layer is able to decrease the nucleation overpotential of Zn and induce uniform Zn^2+^ flux, leading to a dense and smooth Zn deposition. Except for polymers, inorganic nanoparticles can also be used as spacers to induce the Zn uniform deposition. Xu and co-workers reported a zincophilic layer composed of electronic conductive N/Se-doped MXene nanosheets and ionic conductive ZnSe nanoparticles by in-situ construction [95]. The N/Se-MXene@ZnSe mixed conducting film not only provides sufficient and uniform ion-transport channels and decreases the nucleation overpotential of Zn but also avoids the direct contact between Zn anodes and electrolytes, preventing HER and other side reactions. In addition, Ye et al. used tetramethylammonium hydroxide as a surface modifier to realize the TMA^+^ intercalation in MXene during the etching MAX phase [115]. After that, ultrasonic and purification treatment easily obtained ultrapure MX-TMA nanosheet dispersion. MX-TMA protective layer was fabricated through an in-situ spontaneously reducing/assembling strategy. Owing to the abundant zincophilic sites and well hydrophilicity, Zn^2+^ transportation has successfully been promoted to uniform the electrodeposition and inhibits spontaneous side reactions.

Recently, researchers have exploited other properties of spacers to induce smooth Zn^2+^ deposition. Yang et al. fabricated an MXene-mPPy layer that exhibited an exceptional charge enrichment ability, which was beneficial for homogenizing the dispersions of electric field and ion flux in the interfaces between Zn anodes and aqueous electrolytes [97]. Thus, dendrite-free Zn anodes with long-cycle performance were achieved. Moreover, Feng et al. also fabricated a composite protective layer composed of MXene and ZnS, where the low zincophilicity of ZnS promoted the interfacial concentration of Zn^2+^, which was favorable to the homogeneous deposition [93]. These works inspired further research on the MXene interface engineering of Zn anodes.

#### As Host Materials

Compared to planar Zn foil, a 3D anode with a high surface area and an interconnected stereochemical structure favor transformation of Zn^2+^/Zn particles, deposition of Zn metal and electrolyte contact with anodes, contributing to a dendrite-free cycling behavior and high-rate capability. Furthermore, they show shorter ion diffusion routes and more active sites, permitting fast Zn^2+^/Zn redox kinetics. To date, researchers cast their attention on the application of MXene as host materials of Zn anodes in ZIBs owing to the high electronic conductivity, high physical performances, and other characteristics. High electronic conductivity and zincophilicity inhibit dendrite growth and extend cycle life. Secondly, high flexibility and mechanical strength make this material feasible in rigid and flexible batteries. Thirdly, the large specific surface area and deposition space provided by the 3D structure of MXene host materials greatly avoid instability due to the volume change during the Zn deposition and stripping process. For example, Feng et al. fabricated an MXene paper through vacuum filtrating of MXene colloidal solution (Fig. 6c) [116]. The flexible, free-standing, 3D layered MXene paper works as a current collector and anode material for Zn deposition. Compared with Zn foil and Zn wire which were directly used as flexible anodes for ZIBs in early research, MXene paper possesses unlimited flexibility to satisfy the demand of wearable or even bendable applications. Moreover, different from Zn metal suffers from the shape memory effect, MXene paper can withstand continuous bending. Similar methods to modify the MXene protective layers can also be used to modify the MXene framework materials. The same research group designed multifunctional uniform antimony (Sb nanoarrays on MXene paper for dendrite-free Zn anodes [117]. The as-prepared Sb nanoarrays can reversibly alloy with Zn to form ZnSb intermetallic phase and act as zincophilic nucleation sites. Benefiting from the zincophilic alloy and 3D MXene architecture, the MXene@ZnSb can effectively induce smooth Zn deposition and suppress side reactions.

Apart from employing zincophilic particles as spacers, combining other nano-materials with MXene is another appealing tactic. Inspired by the previous research on graphene-protecting metal anodes of metal–ion batteries, Chen and co-workers used an oriented freezing process to create a flexible MXene/graphene scaffold as Zn anode materials (Fig. 6d) [112]. Compared with other 2D nano-materials, MXene possess much higher zincophilicity to induce Zn deposition within the interior of the framework structure instead of its upper surface. Both graphene nanosheets in the MXene framework and oriented freezing technology provide much more sufficient ion-transport channels and Zn storage space to avoid dendrite growth and volume change. Moreover, Chen et al. also reported flexible Ti_3_C_2_T_*x*_/nanocellulose hybrid film as a Zn plating host material [94]. Benefiting from the ultra-low diameter and rich hydroxyl groups of nanocellulose, the hybrid film exhibits better mechanical properties and superior electrolyte wettability, leading to an improved Zn plating/stripping reversibility compared to the pure Ti_3_C_2_T_*x*_-MXene film.

#### As Electrolyte Additives and Separator Modifications

Electrolyte additives and separator modifications are other kinds of anode engineering. During the chare–discharge process, the electrolyte additives shall enrich at the electrode–electrolyte sphere, achieving artificial SEI-like protective interphase by in-situ formation. Meanwhile, the modifications on the one side of the separators shall make complete contact with the Zn anode after assembling batteries, taking effects as an anode protective layer. Wang et al. first used MXene as an electrolyte additive to facilitate uniform Zn deposition by controlling the Zn^2+^ transportation, nucleation, and growth process of Zn (Fig. 6e) [118]. Proper MXene additive concentration can enrich nanosheets at the electrode–electrolyte interface, where the uniform nucleation of Zn^2+^ is well regulated due to the good conductivity and strong binding energy with Zn^2+^ of MXene. Besides, well-dispersed nanosheets perform excellent elasticity, accommodating the surface structure and volume change. Moreover, they synergistically suppressed the dendrites' growth and relieved the side reactions on the Zn anode surface during the continuous plating/striping process. In addition, Feng et al. fabricated a freestanding, lightweight, and zincophilic MXene/nanoporous oxide heterostructure engineered separator, which can homogenize the electric field distribution, promote even Zn ionic flux, regulate uniform Zn deposition, prevent dendrites from penetrating the separator, and suppress side reactions (Fig. 6f) [80].

### Other 2D Materials for Advanced Zn Anodes

**Fig. 7:**
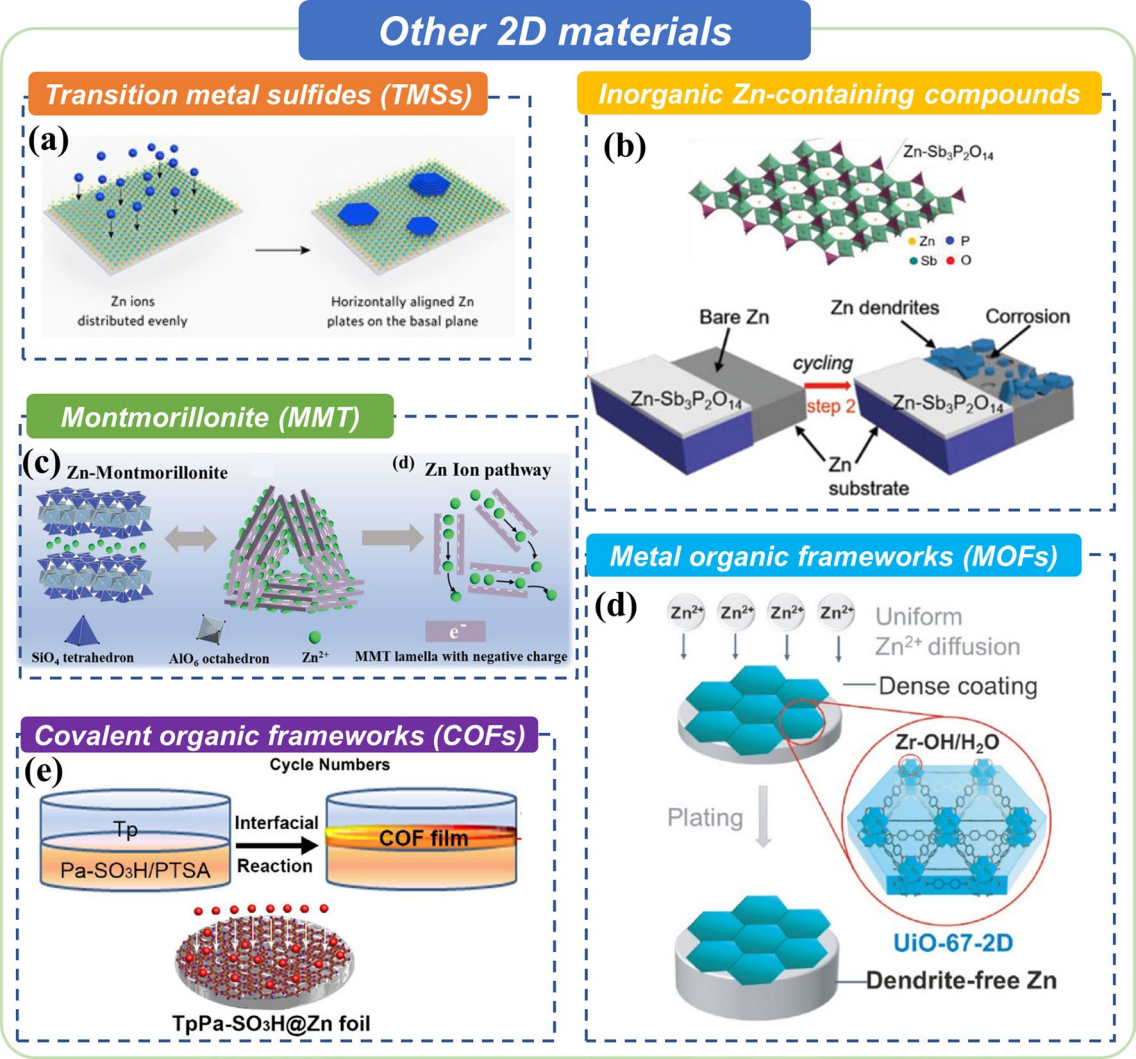
Summary of other 2D materials for advanced Zn anode. **a** Transition metal sulfides (TMSs). Copyright 2022, Wiley. **b** Inorganic Zn-containing compounds. Copyright 2021, Wiley.** c** Montmorillonite (MMT). Copyright 2021, Wiley. **d** Metal organic frameworks (MOFs). Copyright 2022, springer. **e** Covalent organic frameworks (COFs). Copyright 2022, Elsevier

There are still other kinds of 2D materials applied to protect Zn anodes and extend the cycle life of ZIBs. This part shall introduce these materials separately, including transition metal sulfides (TMSs), inorganic Zn-containing compounds, Montmorillonite (MMT), metal–organic frameworks (MOFs), and covalent-organic frameworks (COFs) (Fig. 7).

**Fig. 8:**
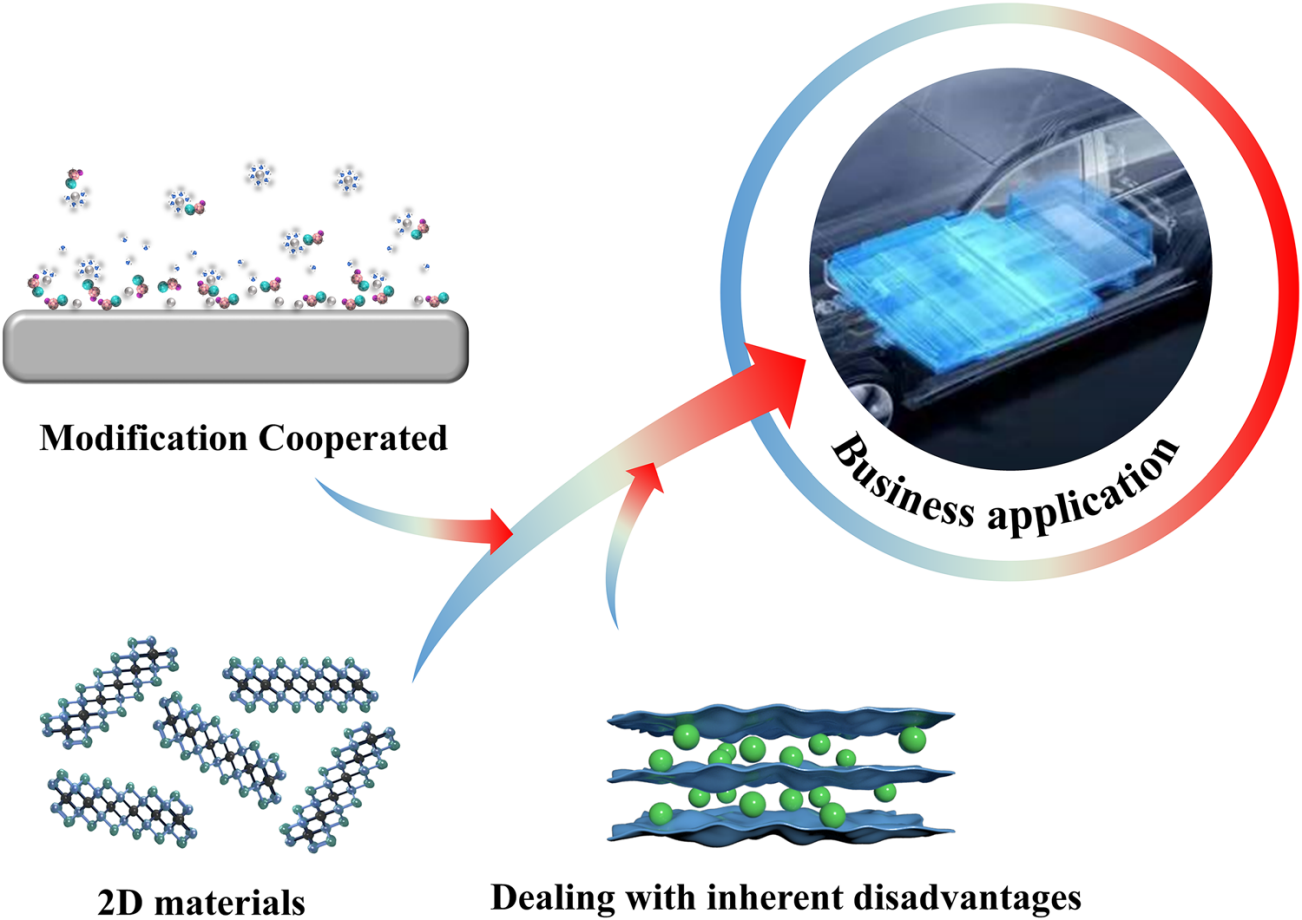
Discovery, evolution, and application of 2D materials in Zn anodes of ZIBs

Molybdenum disulfide (MoS_2_), as a typical kind of TMSs, has been successfully applied to redistribute Zn^2+^ concentration and enhance Zn mono-oriented deposition for stable anode due to its high zincophilicity and low lattice-mismatching. Recently, Alshareef’s group fabricated large-area, mono-orientated MoS_2_ as a substrate to deposit Zn metal (Fig. 7a) [119]. This work designs a complete substrate with a similar lattice structure to guide the non-dendrite deposition of Zn anodes to prolong ZIBs’ cycling life. Moreover, it provides strong evidence to prove the epitaxial growth relationship between the deposited metal and the substrate by comparing the morphology changes during the plating process.

Regarding inorganic Zn-containing compounds, Wu et al. fabricated an artificial protective layer filled with novel 2D Zn^2+^ adsorbed Sb_3_P_2_O_14_^3−^ (Zn–Sb_3_P_2_O_14_) nanosheets, which provided an ideal separation between Zn anode and aqueous electrolyte to suppress the side reactions (Fig. 7b) [120]. Meanwhile, Zn–Sb_3_P_2_O_14_ nanosheets allow Zn^2+^ to pass through the protection layer rapidly. Moreover, the 2D Sb_3_P_2_O_14_^3−^ skeleton with negative charge also confines the 2D diffusion of Zn^2+^ along the lateral surface of the Zn anode, resulting in a uniform electronic deposition.

MMT is a natural phyllosilicate material having a typical layered structure. Each lamella is formed by an alumina octahedral layer sandwiched between two silica tetrahedrons. Due to its unique negative-charged lamella interfaces and positive-charged lamella edges, the interlamellar can act as a freeway for exchangeable cation transportation. Based on the unique feature of MMT, Li et al. developed an artificial Zn ion-conductive SEI on the surface of a Zn metal anode to promote the electrochemical performance of aqueous ZIBs (Fig. 7c) [121]. The obtained interphase exhibits a dense and smooth morphology, which can cut off the Zn anode from the electrolyte to avoid side reactions; thus, Zn^2+^ can migrate into the interspace of the lamella driven by the electric field. Moreover, such a conductive cation film boosts fast Zn^2+^ transport and a high Zn^2+^ transfer number, resulting in a low and stable polarization voltage and an ultralong lifespan of ZIBs.

MOFs are porous crystalline materials constructed from inorganic metal nodes and organic linkers. Compared with other organic and inorganic materials, such as organic polymers and metal oxides, MOF exhibits the following merits in stabilizing Zn metal anodes. Firstly, the intrinsic pore of MOF allows facile diffusion of Zn^2+^, while their insulating nature ensures the deposition of Zn under the MOF-protective layers. Secondly, the surface structure of MOF can be judiciously tuned to regulate the interaction between the Zn surface and electrolytes. The precisely defined crystal structure of MOF further facilitates the understanding of the structure–property relationship to guide the rational design of Zn coating. Thirdly, the morphology of MOF can be controlled to form stable 2D film attaching to the Zn surface. For example, Yuan et al. coated 2D MOF nanosheets on the surface of Zn anode, dramatically reducing the polarization, elongating the cycling lifetime, and showing a low hydrogen evolution (Fig. 7d) [122].

As a fast-developing field of crystalline porous material, COFs are regarded as promising ion transport media due to their structural diversity, high porosity, tunable functionality, and rich physicochemical features. These features have attractive applicability in energy storage devices. Moreover, COFs have functional groups that can provide fast Zn^2+^ transport channels and well-distributed nucleation sites. For example, Zhao’s group developed an interfacial reaction strategy to deposit a COF film based on cation-conducting sulfonic acid on the Zn anode surface (Fig. 7e) [123]. The protective layer effectively prevents Zn^2+^ from accumulation, therefore promoting smooth plating. Moreover, the zincophilic functional groups also facilitate the desolvation of the solvated cation shell, preventing Zn anode from HER and other side reactions.

## Conclusions and Perspectives

In summary, each kind of 2D material possesses different properties, including high specific surface area, abundant surface functional groups and open 2D ion transport channel, which help researchers select specific 2D materials for performance improvement according to different needs. Therefore, they can not only serve as the surface protective layers or host materials of Zn anodes but also act as the additives of separators or electrolytes for advanced Zn anodes. On the one hand, these anode-protective strategies provide a highly specific surface area with many active sites for Zn^2+^ to deposit, resulting in limited dendrite growth. In the case of electronic conductivity for averaging electric fields, 2D materials, especially graphene and MXenes, are the promising candidates. Graphene and MXenes can be assembled into 3D frameworks as host materials to enhance electron separation and ion transportation in anodes. In addition, graphene and MXenes can be fabricated on Zn anodes as artificial interface layers or constructed into conductive substrates to avoid charge accumulation and balance the surface charge distribution, leading to smooth Zn deposition.

Aside from prolonging the cycle life of ZIBs, 2D materials can also improve practicability. Compared with other active metals, Zn is more stable in aqueous electrolytes; therefore, most aqueous ZIBs use Zn foil as their anodes. However, Zn foil with high thickness will undoubtedly lead to a mismatch with cathodes, causing low depth of discharge (DOD), inevitable waste of materials, and low energy density for whole batteries. Unfortunately, the cycle life of ZIBs shall seriously decrease while using thin Zn foil due to a quick consumption of Zn caused by the issues of HER, passivation, and low efficiency of Zn plating/stripping. But 2D materials can successfully solve these problems. Firstly, the flexibility of 2D materials makes them easy to stack as self-supporting membranes, assemble as few-layer interphase or uniformly separate into electrolytes, which significantly increases their application area. While working as an anode substrate or framework materials, Zn deposition can be well controlled to improve DOD and energy density. As for protective layer, separator, or electrolyte additives, 2D materials can reduce the consumption of Zn, improving the practicability of thin Zn foil to achieve a better performance of ZIBs.

Although significant progress has been achieved in 2D materials for applications in aqueous ZIB anodes, several challenges still need further consideration.

Firstly, 2D materials are tended to restack into densely packed structures or films due to their van der Waals forces between the neighboring layers while the stacking process, resulting in significantly decreased active surfaces and long ion transport pathways. Ordered and controllable microstructures have to be further considered in the design of 2D materials. Various metal ions, organic molecules, and other particles can be introduced into the interlayers of these 2D active materials as spacers to strengthen their structural stability. Fortunately, some 2D materials like MXene and graphene easily graft with other functional groups or molecules, which allows more options for improving properties.

Secondly, some artificial interface layers and substrates based on 2D materials have been designed to overcome the dendrite issue. However, there is still a need for more efforts to improve overall performance. For example, owing to the huge volume change of Zn anodes, the 2D artificial interface layers usually suffer severe structural damages during the repeated Zn plating/stripping process, leading to unsatisfied cycling behavior. Therefore, artificial interface layers and more flexible and tough substrates should be designed. In addition, the network size of the 2D conductive substrates plays an essential role in the Zn electrodeposition. The networks with narrow channels will not be beneficial for the accommodation of electro-deposited large Zn sheets in the charging process, while the networks with too large sizes result in the waste of space in conductive substrates. Thus, further optimization of the frameworks and pore structures of 2D conductive substrates will be significant for high-performance Zn anodes.

Thirdly, a single strategy was often used to protect Zn anodes, resulting in an uncomprehensive development, making ZIBs difficult to implement. Synergistic effects with various Zn anode protection strategies, including introducing functional additives into artificial interface layers and 3D conductive substrates for Zn anode, employing electrolyte additions or “water in salt” electrolytes, could be considered for the further improvement in ZIBs. Besides, considering 2D material itself, the practical application in ZIBs is mainly limited by the complicated synthesis process and high cost of 2D materials; therefore, developing new materials and better manufacturing technologies is urgently needed. Also, the exploration of the 2D materials in Zn anodes is mostly focused on the coin cells, which deviates from actual needs like large capacity and flexible batteries. In addition, the mass production of 2D materials-based anodes on a large scale remains challenging. The future study should not only focus on reducing the cost of synthesizing 2D material; more importantly, the manufacturing process of the 2D materials-based anodes needs to be simplified, thus further reducing the cost to meet industrial standards.

Last but not least, the mechanism of 2D materials still requires intensive study. For example, the phenomenon that a suitable substrate can effectively suppress dendrite growth has already been discovered since 2019 [77]; however, the explanation about epitaxial growth still lacks strong evidence to prove the relationship between deposited metal and substrate [119]. In-situ characterization techniques like the in-situ optical microscope (OM), in-situ X-ray diffraction (XRD), in-situ scanning electron microscope (SEM), in-situ atomic force microscope (AFM) and other related technologies need to be taken more seriously in this area to help researchers get a much clearer understanding of the deposition-stripping process.

Overall, emerging 2D materials for metal anode protection have become research hotspots (Fig. 8). With the rapid development, it is sure that more and more 2D materials will be applied in the modification work of Zn anodes in ZIBs in the future. However, there is still a long way to go before ZIBs can be put into practical applications.
